# Pemigatinib in previously treated Chinese patients with locally advanced or metastatic cholangiocarcinoma carrying *FGFR2* fusions or rearrangements: A phase II study

**DOI:** 10.1002/cam4.5273

**Published:** 2022-09-20

**Authors:** Guo‐Ming Shi, Xiao‐Yong Huang, Tian‐Fu Wen, Tian‐Qiang Song, Ming Kuang, Hai‐Bo Mou, Le‐Qun Bao, Hai‐Tao Zhao, Hong Zhao, Xie‐Lin Feng, Bi‐Xiang Zhang, Tao Peng, Yu‐Bao Zhang, Xiang‐Cheng Li, Hong‐Sheng Yu, Yu Cao, Lian‐Xin Liu, Ti Zhang, Wei‐Lin Wang, Jiang‐Hua Ran, Ying‐Bin Liu, Wei Gong, Ming‐Xia Chen, Lian Cao, Yang Luo, Yan Wang, Hui Zhou, Guo‐Huan Yang, Jia Fan, Jian Zhou

**Affiliations:** ^1^ Liver Surgery and Transplantation, Zhongshan Hospital Fudan University Shanghai China; ^2^ Hepatobiliary Surgery, West China Hospital Sichuan University Chengdu China; ^3^ Hepatobiliary Surgery Tianjin Cancer Hospital Tian Jin China; ^4^ Department of Oncology, Hepatobiliary and Pancreatic Surgery Center The First Affiliated Hospital of Sun Yat‐sen University Guangzhou China; ^5^ Medical Oncology Shulan (Hangzhou) Hospital Hangzhou China; ^6^ Hepatobiliary Surgery Hubei Cancer Hospital Wuhan China; ^7^ Liver Surgery Peking Union Medical College Hospital Beijing China; ^8^ Hematological Surgery Department Cancer Hospital of Chinese Academy of Medical Science Beijing China; ^9^ Hepatobiliary and Pancreatic Surgery Sichuan Cancer Hospital Chengdu China; ^10^ Hepatobiliary Surgery, Tongji Hospital Tongji Medical College of HUST Wuhan China; ^11^ Hepatological Surgery Department The First Affiliated Hospital of Guangxi Medical University Nanning China; ^12^ Hepatobiliary and Pancreatic Surgery Cancer Hospital Affiliated to Harbin Medical University Harbin China; ^13^ Liver Surgery Jiangsu Province Hospital Nanjing China; ^14^ Oncology Department Affiliated Hospital of Qingdao University Qingdao China; ^15^ Phase 1 Clinical Research Center Affiliated Hospital of Qingdao University Qingdao China; ^16^ Hepatobiliary Surgery Department The First Affiliated Hospital of University of Science and Technology of China Hefei China; ^17^ Hepatobiliary Surgery Department Tianjin Cancer Hospital Tianjin China; ^18^ Hepatopancreatobiliary Surgery The Second Affiliated Hospital of Zhejiang University School of Medicine Hangzhou China; ^19^ Hepatopancreatobiliary Surgery The First Hospital of Kunming Kunming China; ^20^ General Surgery Xin Hua Hospital Affiliated to Shanghai Jiao Tong University School of Medicine Shanghai China; ^21^ Department of Medical Science and Oncological Strategy Innovent Biologics Inc. Suzhou China

**Keywords:** antitumor activity, cholangiocarcinoma, *FGFR2* fusions or rearrangements, pemigatinib, phase II

## Abstract

**Objective:**

This study evaluated the antitumor activity and safety of pemigatinib in previously treated Chinese patients with advanced cholangiocarcinoma and fibroblast growth factor receptor 2 (*FGFR2*) fusions or rearrangements.

**Background:**

Pemigatinib provided clinical benefits for previously treated patients with cholangiocarcinoma carrying *FGFR2* fusions or rearrangements and was approved for this indication in multiple countries.

**Methods:**

In this ongoing, multicenter, single‐arm, phase II study, adult patients with locally advanced or metastatic cholangiocarcinoma carrying centrally confirmed *FGFR2* fusions or rearrangements who had progressed on ≥1 systemic therapy received 13.5 mg oral pemigatinib once daily (3‐week cycle; 2 weeks on, 1 week off) until disease progression, unacceptable toxicity, or consent withdrawal. The primary endpoint was objective response rate (ORR) assessed by an independent radiology review committee.

**Results:**

As of January 29, 2021, 31 patients were enrolled. The median follow‐up was 5.1 months (range, 1.5–9.3). Among 30 patients with *FGFR2* fusions or rearrangements evaluated for efficacy, 15 patients achieved partial response (ORR, 50.0%; 95% confidence interval [CI], 31.3–68.7); 15 achieved stable disease, contributing to a disease control rate of 100% (95% CI, 88.4–100). The median time to response was 1.4 months (95% CI, 1.3–1.4), the median duration of response was not reached, and the median progression‐free survival was 6.3 months (95% CI, 4.9–not estimable [NE]). Eight (25.8%) of 31 patients had ≥grade 3 treatment‐emergent adverse events. Hyperphosphatemia, hypophosphatasemia, nail toxicities, and ocular disorders were mostly <grade 3, except for 2 events ≥grade 3.

**Conclusions:**

The encouraging antitumor activity and favorable safety profile support the use of pemigatinib as a treatment in previously treated Chinese patients with cholangiocarcinoma and *FGFR2* rearrangements.

## 1 INTRODUCTION

Cholangiocarcinoma is a cluster of heterogenous and aggressive tumors that originate from the biliary tract, which can be classified into intrahepatic, perihilar, and distal cholangiocarcinoma based on the anatomical location.^1^ Though considered to be a relatively rare cancer occurring in <6 per 100,000 people in most countries, cholangiocarcinoma has aroused concern because of an increase in incidence in most countries from 1993 to 2012.^2^ China had the second‐largest increase in the incidence of intrahepatic cholangiocarcinoma (ICC) during this period, with an average annual percentage change of 11.1%,^2^ and risk factors such as hepatitis B or hepatitis C infections may have contributed to the rise.^3^ The prognosis of cholangiocarcinoma is poor due to late‐stage diagnosis and limited treatment options, with an estimated 5‐year survival of 7%–20%.^4^ The mortality rate of cholangiocarcinoma varies by racial or ethnic groups and is higher in Asian populations and American Indian and Alaska Natives than in other populations.^5^

Approved systemic treatments for unresectable locally advanced or metastatic cholangiocarcinoma are limited to chemotherapies in mainland China. The first‐line standard of care for locally advanced or metastatic cholangiocarcinoma is gemcitabine plus cisplatin^6^, ^7^; however, this offers limited benefit as most patients will experience disease progression within 1 year of treatment.^8^ FOLFOX regimen (fluorouracil plus leucovorin and oxaliplatin) is a preferred subsequent treatment for cholangiocarcinoma,^6^ but this regimen and other subsequent chemotherapies provide modest survival benefits, with a median overall survival (OS) of <1 year, and are coupled with frequent high‐grade toxicity in clinical trials^9^, ^10^ and real‐world situations.^11^

Patients with cholangiocarcinoma carrying *FGFR2* fusions or rearrangements represent a distinct genetic subgroup, associated with specific histopathological phenotypes and prognoses.^12^, ^13^ *FGFR2* rearrangements occur almost exclusively in ICC at an estimated frequency of 10%–16% in studies conducted in other countries^12^, ^13^, ^14^ and 6.6%–20% among Chinese patients.^15^, ^16^, ^17^ FGF/FGFR signaling pathways act as critical regulators of cell survival, cell proliferation, migration, and angiogenesis, and *FGFR2* translocations are linked in neoplastic transformation.^18^, ^19^ *FGFR2* rearrangements are clinically actionable genetic alterations, as supported by evidence of clinical benefits with targeting the FGF/FGFR signaling pathways in patients with cholangiocarcinoma containing *FGFR2* rearrangements.^20^

Pemigatinib is an oral, potent, and selective inhibitor of FGFR1, FGFR2, and FGFR3.^21^ It is the first targeted treatment approved for cholangiocarcinoma in the United States, the European Union, Japan, and Taiwan (China), indicated for previously treated cholangiocarcinoma with an *FGFR2* fusion or rearrangement.^22^, ^23^, ^24^, ^25^ The clinical benefits and manageable safety profile of pemigatinib in patients with cholangiocarcinoma carrying *FGFR2* fusions or rearrangements were demonstrated in the FIGHT‐202 study, which was a single‐arm, phase II study that enrolled patients with *FGFR2* fusions or rearrangements (*n* = 107), other *FGF/FGFR* alterations (*n* = 20), and no *FGF/FGFR* alterations (*n* = 18) from multiple countries, not including mainland China.^26^ In patients with cholangiocarcinoma carrying *FGFR2* fusions or rearrangements, pemigatinib 13.5 mg once daily resulted in an objective response rate (ORR) of 35.5%, with a median duration of response (DOR) of 7.5 months, and a disease control rate (DCR) of 82.2%. Median progression‐free survival (PFS) was 6.9 months and median OS was 21.1 months. Pemigatinib was less effective in patients with other or no *FGF/FGFR* alterations, but was well tolerated in all patient cohorts.^26^

This phase II trial was a bridging study to assess whether the efficacy and safety findings for pemigatinib in FIGHT‐202 could be extrapolated to previously treated Chinese patients with cholangiocarcinoma carrying *FGFR2* fusions or rearrangements. We also report results from a molecular epidemiology study of *FGFR2* rearrangements in Chinese patients with cholangiocarcinoma.

## 2 PATIENTS AND METHODS

### 2.1 Study design and patients

This ongoing, multicenter, single‐arm, phase II study was conducted at 14 hospitals in China. The study was registered on ClinicalTrials.gov (NCT04256980). Patients received 13.5 mg oral pemigatinib once daily in 3‐week cycles with 2 weeks on and 1 week off until disease progression, unacceptable toxicity, or withdraw of consent.

Eligible patients were ≥18 years old, had histologically or cytologically confirmed locally advanced or metastatic cholangiocarcinoma with centrally confirmed *FGFR2* fusions or rearrangements, and had progressed on at least one prior systemic therapy. Patients must have had at least one measurable lesion according to Response Evaluation Criteria in Solid Tumors (RECIST) v1.1, an Eastern Cooperative Oncology Group (ECOG) performance status of 0 or 1, and a survival expectancy of ≥12 weeks. Key exclusion criteria included prior treatment with a selective FGFR inhibitor, history of calcium and phosphate hemostasis disorder, or systemic mineral imbalance with ectopic calcification of soft tissue (with the exception of calcifications of skin, kidney, tendons, or blood vessels due to injury, diseases, and aging, in the absence of systemic mineral imbalance), clinically significant corneal or retinal disorder confirmed by ophthalmologic examination, and use of any potent CYP3A4 inhibitors or inducers within 14 days or 5 half‐lives, whichever is shorter, before the first dose of study drug (with the exception of topical ketoconazole).

This study was accompanied by a retrospective, multicenter, molecular epidemiology study on the frequency of *FGFR2* rearrangements in Chinese patients with ICC. ICC tissue samples from tissue repositories at 13 clinical sites (Table S1) were included if patients were ≥18 years old and the tissues had been stored in the repositories for ≤5 years. Patients in the molecular epidemiology study received standard therapies.

The studies were conducted in accordance with the principles of the Declaration of Helsinki, Good Clinical Practice guidelines, and local applicable regulatory requirements. The study protocols and informed consent were approved by independent ethics committees at participating sites. All participants signed written informed consent forms.

### 2.2 Endpoints and assessments

Patients in the phase II study were prescreened for *FGFR2* status by next‐generation sequencing (NGS) with the Malignant Neoplasms Multi‐Gene Analysis Kit (Suzhou Geneplus Biomedical Engineering Co., Suzhou, China) at the central laboratory on archival formalin‐fixed and paraffin‐embedded tumor samples or fresh tumor samples; *FGFR2* status reports based on DNA sequencing performed within 2 years could also be used; a relative abundance of variant allele of >5.3% was used to define *FGFR2* fusions or rearrangements (the lower boundary of frequency detected by FMI central laboratory in FIGHT‐202 study). The primary endpoint of the phase II study was ORR (complete response [CR] or partial response [PR]), as assessed by an independent radiology review committee (IRRC) according to RECIST v1.1. Secondary endpoints included investigator‐assessed ORR (per RECIST v1.1), PFS (time from first dose of study treatment to the first documented progressive disease [PD] or death due to any cause), DOR (time from first documented CR or PR until PD or death due to any cause), DCR (CR, PR, or stable disease [SD]), OS (time from first dose of study treatment to the first documented death due to any cause), time to response (TTR; time from first dose of study treatment to the first documented CR or PR), safety, and tolerability. Tumor response was assessed by computed tomography or magnetic resonance imaging at baseline, every 6 weeks until week 12, and every 9 weeks thereafter until discontinuation of treatment, PD, death, or completion of the study. Safety was monitored up to 30 days after discontinuation of treatment. Safety assessments included adverse events, vital signs, 12‐lead electrocardiograms, physical examinations, eye examinations, and clinical laboratory tests (including serum calcium, serum phosphate, 25‐hydroxyvitamin D, and parathyroid hormone). Adverse events were graded according to National Cancer Institute Common Terminology Criteria for Adverse Events v5.0. Treatment‐emergent adverse events (TEAEs; defined as an adverse event that emerged or worsened from first dose of treatment to 30 days after discontinuation of treatment) are reported. Sponsor‐defined clinically notable TEAEs included hyperphosphatemia, hypophosphatasemia, nail toxicities, and retinal detachment due to subretinal fluid accumulation.

The objective of the molecular epidemiology study was to determine the frequency of *FGFR2* rearrangements in Chinese patients with ICC. *FGFR2* rearrangements were detected by fluorescence in situ hybridization (FISH) with a break‐apart FISH probe kit (Amoy Diagnostics) in archival formalin‐fixed and paraffin‐embedded tumor samples.

### 2.3 Statistical analyses

Assuming an ORR of 35%, 26 patients were estimated to provide the phase II study with a probability of >80% to detect an ORR of ≥26.5% (the lower limit of the 95% confidence interval [CI] for ORR in the FIGHT‐202 trial^26^) and a 95% CI with a lower limit ≥11%; to account for a dropout rate of 20%, 33 patients were planned to be enrolled. It was considered appropriate to extrapolate results from FIGHT‐202 to Chinese patients if this study met the predefined threshold of positive results and did not show any unexpected major safety concerns.

Efficacy endpoints were analyzed primarily in the efficacy‐evaluable population (EEP), which included patients with centrally confirmed *FGFR2* rearrangements who received at least one dose of pemigatinib. Efficacy was also evaluated in the per‐protocol set (PPS), which included patients in the EEP without protocol violations. Safety was assessed in all patients who received at least one dose of pemigatinib (safety set [SS]).

Data were summarized using descriptive statistics. The 95% CIs for ORR and DCR were estimated using the Clopper‐Pearson method. Time‐to‐event data were analyzed using the Kaplan–Meier method. The frequency of *FGFR2* rearrangements in ICC was summarized by the percentage of patients with confirmed *FGFR2* rearrangements among all patients included in the molecular epidemiology study.

## 3 RESULTS

### 3.1 Patients

**FIGURE 1:**
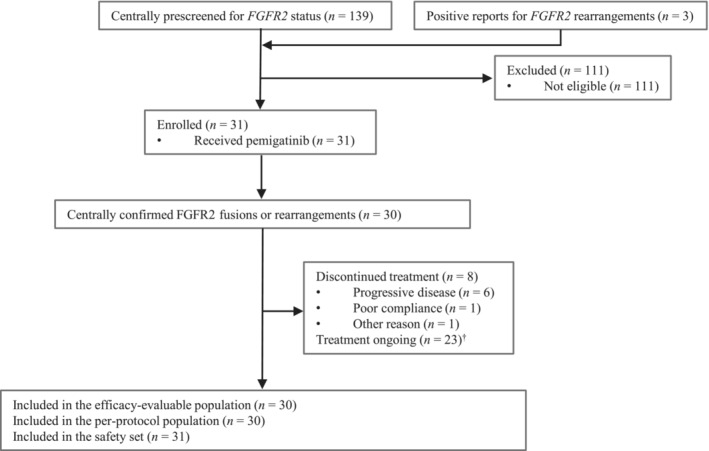
Trial profile. ^†^At analysis cutoff date (January 29, 2021).

Between March 3, 2020, and December 15, 2020, 139 patients, including 11 subjects who had positive genetic results in the third‐party commercial reports and 10 other subjects who had detectable *FGFR2* rearrangements from the molecular epidemiology study, were assessed for *FGFR2* status in the phase II study by central laboratory, and three patients who already had previous positive reports for *FGFR2* rearrangements based on DNA sequencing by Geneplus were also screened (Figure 1). After exclusion of 111 patients who did not meet the eligibility criteria, 31 patients were enrolled and received at least one dose of pemigatinib (included in the SS). Among them, 30 patients with centrally confirmed *FGFR2* fusions or rearrangements were included in the EEP and PPS, and one patient was excluded due to relative abundance of *FGFR2* rearrangements lower than the prespecified threshold.

As of the data cutoff date of January 29, 2021, eight patients discontinued treatment, with PD being the most common reason, and 23 patients were still receiving treatment. The median duration of treatment was 3.8 months (range, 1.5–7.4). The median duration of follow‐up was 5.1 months (range, 1.5–9.3) in the EEP/PPS.

Of 31 patients enrolled, most were female (67.7%), the median age was 56 years, and most had a diagnosis of ICC (96.8%) and stage IV disease (90.3%). Fifteen (48.4%) patients had a history of hepatitis, and 22 (71.0%) had a positive test for hepatitis B surface antigen (HBsAg) or hepatitis B core antibody (HBcAb; Table 1). About half of the patients received ≥2 lines of therapy. Most patients had received chemotherapy (96.8%), and some patients had also received other types of prior antitumor treatments such as immunotherapy (41.9%), targeted therapy (32.3%), and radiotherapy (25.8%).

**TABLE 1:** Patient baseline characteristics

Characteristic	All patients (*N* = 31)
Age, median (range), years	56 (28–68)
Gender	
Male	10 (32.3)
Race	
Asian	31 (100)
ECOG performance status	
0	16 (51.6)
1	15 (48.4)
Cholangiocarcinoma location	
Intrahepatic	30 (96.8)
Perihilar	1 (3.2)
Adenocarcinoma	30 (96.8)
TNM stage IV	28 (90.3)
History of hepatitis B	15 (48.4%)
HBsAg‐ or HBcAb‐positive	22 (71.0)
Number of previous systemic therapies for advanced metastatic disease	
1	16 (51.6)
2	8 (25.8)
≥3	7 (22.6)
Type of previous antitumor therapy	
Chemotherapy	30 (96.8)
Surgery	19 (61.3)
Immunotherapy	13 (41.9)
Targeted therapy	10 (32.3)
Radiotherapy	8 (25.8)
Other therapy	10 (32.3)

*Note*: Data are *n* (%) unless otherwise stated.

Abbreviation: ECOG, Eastern Cooperative Oncology Group.

### 3.2 Frequency of FGFR2 rearrangements and fusion partners among Chinese patients with ICC

In the molecular epidemiology study, 728 patients with ICC were included and 717 samples had readout as of October 31, 2020. Among them, 44 (6.14%, 44/717) patients had detectable *FGFR2* rearrangements by FISH. The proportion of patients with *FGFR2* rearrangements by study site is presented in Table S1. The prevalence of *FGFR2* rearrangements was highest in Southwest China (Sichuan and Yunnan province, 10.5%) and lowest in South China (Guangdong and Guangxi province, 5%).

Based on NGS results from 30 patients with confirmed *FGFR2* rearrangements in the phase II study, 26 *FGFR2* fusion partners were identified (Table S2). Most of the fusion partners were unique to individual patients (22 of 30). The most common fusion was *FGFR2*‐*WAC*, identified in three (10.0%) patients.

### 3.3 Efficacy

**FIGURE 2:**
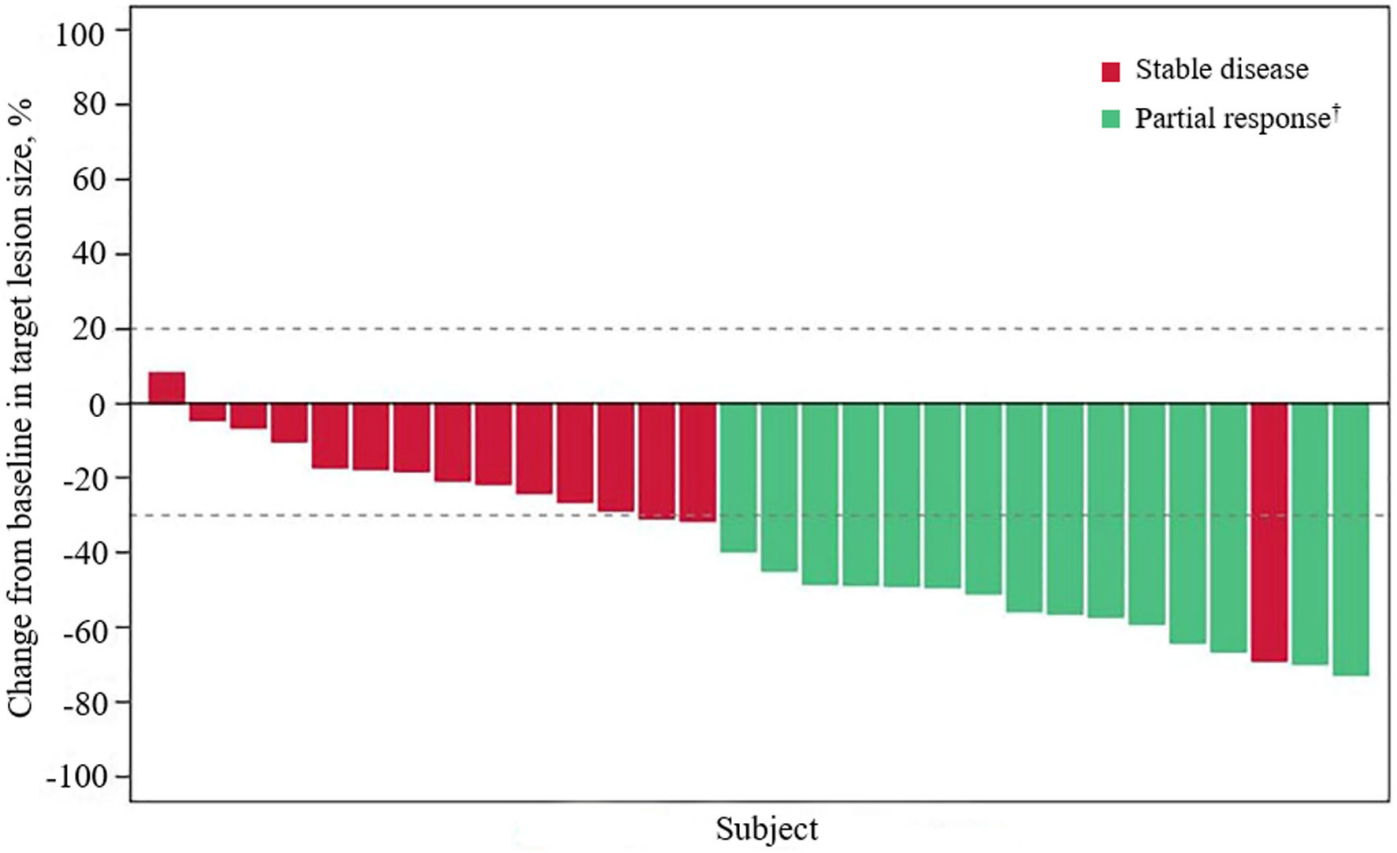
Best percentage change from baseline in target lesion size for individual patients carrying FGFR2 rearrangements. ^†^Green bar indicates confirmed responses assessed by an independent radiology review committee (IRRC) according to RECIST v1.1. Abbreviation: FGFR2, fibroblast growth factor receptor; RECIST 1.1, Response Evaluation Criteria in Solid Tumors version 1.1.

**FIGURE 3:**
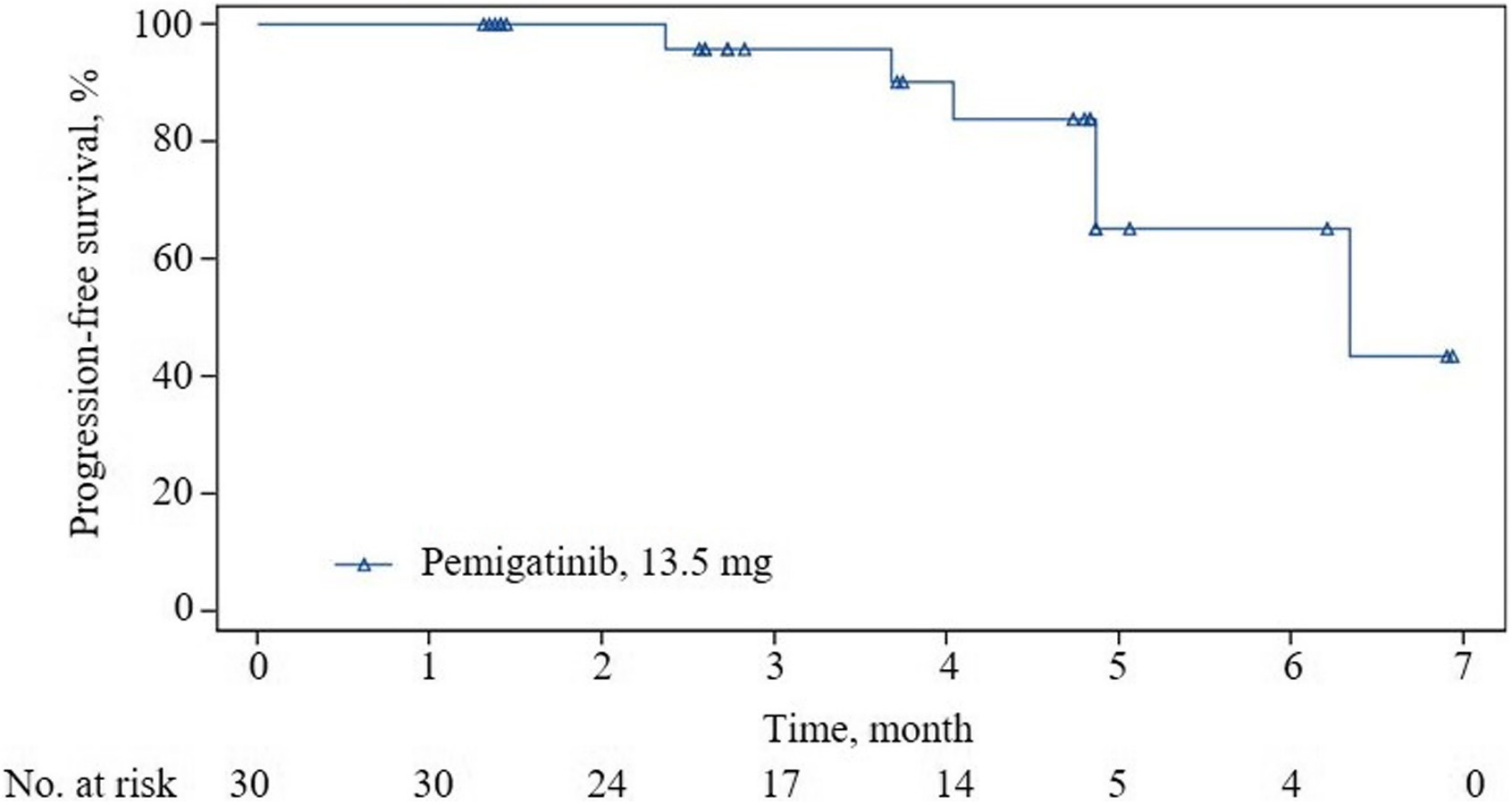
Kaplan–Meier estimates of progression‐free survival

Fifteen patients (ORR, 50.0%; 95% CI, 31.3–68.7) achieved a confirmed objective response according to IRRC assessments, all being PR; the data met the prespecified criteria of positive results (Figure 2 and Table 2). The median TTR was 1.4 months (95% CI, 1.3–1.4), and the median DOR was not reached (95% CI, 3.4–NE). Most patients (29 [96.7%]) had an IRRC‐assessed reduction from baseline in target lesion size. Additionally, 15 (50%) patients achieved SD, contributing to a DCR of 100% (95% CI, 88.4–100). As of the data cutoff, six patients had PD (*n* = 5) or died (*n* = 1; the patient died 77 days after the last dose of pemigatinib due to PD). Median PFS was 6.3 months (95% CI, 4.9–NE) according to IRRC assessments (Figure 3 and Table 2).

**TABLE 2:** Primary and secondary efficacy endpoints

Efficacy outcomes	EEP/PPS (*N* = 30)
Objective response rate	15 (50), 31.3–68.7
Complete response	0
Partial response	15 (50.0%)
Stable disease	15 (50.0%)
Progressive disease	0
Disease control rate	30 (100), 88.4–100
Time to response, median, months	1.4 (1.3–1.4)
Duration of response	
Median, months	NR (3.4–NE)
At 3 months	100 (100–100)
Progression‐free survival	
Median, months	6.3 (4.9–NE)
At 3 months	95.8 (73.9–99.4)
At 6 months	65.1 (33.2–84.7)
Overall survival	
Median, months	NR (NE)
At 3 months	96.0 (74.8–99.4)
At 6 months	96.0 (74.8–99.4)
At 9 months	96.0 (74.8–99.4)

*Note*: Data are *n* (%), 95% CI; months (95%); % 95% CI. Tumor response was assessed by an independent radiology review committee according to RECIST v1.1.

Abbreviations: CI, confidence interval; EEP, efficacy‐evaluable population; PPS, per‐protocol set; NE, not evaluable; NR, not reached; RECIST 1.1, Response Evaluation Criteria in Solid Tumors version 1.1.

The tumor response to pemigatinib was also confirmed by investigator assessments (Table S3). The investigator‐assessed ORR (40%; 95% CI, 22.7–59.4) also met the prespecified criteria of positive results. Median PFS was not reached (95% CI, 4.9–NE) according to investigator assessments.

OS data were not mature at data cutoff. One patient died. The median OS was not reached (95% CI, NE; Table 2 and Figure S1).

### 3.4 Safety

All patients experienced at least one TEAE (Table 3). The most common TEAEs were hyperphosphatemia (77.4%), dry mouth (54.8%), and alopecia (54.8%). Eight (25.8%) patients had ≥grade 3 TEAEs, each occurring in one patient except for hypercalcemia (two [6.5%]); there were no grade 5 TEAEs. Serious TEAEs occurred in three (9.7%) patients, which were rectum polyps, abnormal liver function, and biliary tract infection. No patients discontinued treatment due to TEAEs. One (3.2%) patient had a dose reduction due to blurred vision, and four (12.9%) patients had dose interruption due to hypercalcemia, pain in extremity, abnormal liver function, and vomiting, respectively.

**TABLE 3:** Common treatment‐emergent adverse events

	Safety set (*N* = 31)	
Treatment‐emergent adverse events	Any grade	Grade 3–4
Any treatment‐emergent adverse events	31 (100)	8 (25.8)
Hyperphosphatemia	24 (77.4)	1 (3.2)
Dry mouth	17 (54.8)	0
Alopecia	17 (54.8)	0
Diarrhea	13 (41.9)	0
Fatigue	11 (35.5)	0
Dysgeusia	10 (32.3)	0
Arthralgia	10 (32.3)	0
Nail detachment	10 (32.3)	0
Decreased appetite	8 (25.8)	0
Nail discoloration	8 (25.8)	0
Mucositis oral	7 (22.6)	0
Aspartate aminotransferase increased	7 (22.6)	0
Alanine aminotransferase increased	6 (19.4)	0
Blood phosphatase increased	6 (19.4)	0
Hypophosphatemia	6 (19.4)	0
Platelet count decreased	6 (19.4)	0
Blurred vision	6 (19.4)	1 (3.2)
Abdominal pain	5 (16.1)	0
Constipation	5 (16.1)	0
Trichiasis	5 (16.1)	0
Dry eye	5 (16.1)	0
White blood cell decreased	5 (16.1)	0
Corneal abrasion	5 (16.1)	0
Blood bilirubin increased	4 (12.9)	0
Palmar‐plantar erythrodysesthesia	4 (12.9)	0
Conjunctivitis	4 (12.9)	0
Pain in extremity	4 (12.9)	1 (3.2)
Blood parathyroid hormone decreased	4 (12.9)	0

*Note*: Data are *n* (%). Any grade adverse events reported in at least 10% of patients and all grade 3–4 events are shown.

Among sponsor‐defined clinically notable TEAEs, hyperphosphatemia (median time to onset, 8 days [range, 5–117]) occurred in 24 (77.4%) patients, among whom one (3.2%) was ≥grade 3. The other sponsor‐defined clinically notable TEAEs were all <grade 3 except for blurred vision (grade 3, one [3.2%]). Nail toxicities (median time to onset, 58.5 days [range, 9–138]) were reported in 16 (51.6%) patients, and hypophosphatasemia (median time to onset, 54 days [range, 23–134]) occurred in seven (22.6%) patients, and retinal detachment due to subretinal fluid accumulation (median time to onset, 22 days [range, 21–30]) was found in three (9.7%) patients.

Mean blood phosphate among patients in the SS increased above baseline in the first cycle of treatment (increased by 0.84 mmol/L on day 8 and by 1.08 mmol/L on day 15), which decreased to below baseline from cycle 2 to the end of treatment (reduced by 0.03–0.33 mmol/L; Figure S2). Mild increases in mean blood calcium were observed, with most patients experiencing grade 1 increase in blood calcium. The changes in blood 25‐hydroxyvitamin D were modest for most patients during treatment. There was a trend of decrease in mean parathyroid hormone level over time.

## 4 DISCUSSION

In this phase II bridging study of pemigatinib in previously treated Chinese patients with unresectable locally advanced or metastatic cholangiocarcinoma carrying *FGFR2* fusions or rearrangements, half of the patients achieved a durable objective response, and the other half achieved stable disease. Most TEAEs were mild to moderate and manageable, and no new safety signals were noted. Based on the positive results, the efficacy and safety findings in the multinational FIGHT‐202 study are likely to apply to Chinese patients. This is the first study providing evidence for the efficacy and safety of pemigatinib among Chinese patients with cholangiocarcinoma carrying *FGFR2* fusions or rearrangements.

The frequency of *FGFR2* rearrangements in ICC found in our epidemiology study (6.14%) was slightly lower than that found in FIGHT‐202 and other studies conducted in foreign countries (9%–16%),^12^, ^13^, ^14^, ^26^, ^27^, ^28^ but consistent with that found in studies of Chinese patients (5.2%–20%).^15^, ^16^, ^17^, ^29^, ^30^ Based on the prevalence of *FGFR2* rearrangements, pemigatinib would provide clinical benefits to a great number of Chinese patients with ICC. Given the large sample size (728 in our study vs. 40–257 for ICC in^15^, ^16^, ^17^, ^29^, ^30^), our epidemiology study may offer a more reliable estimate of *FGFR2* rearrangement frequency in Chinese patients with ICC. Consistent with previous studies, *FGFR2* fusion partners identified in Chinese patients with ICC in our study are diverse and mostly individual‐specific; 26 fusion partners were identified in Chinese patients as compared with up to 128 in other populations.^31^ Relatively common partners such as *B1CC* and *AHCYL1* were also seen in our patient cohort.^18^, ^26^ *B1CC* was the most common fusion partner in the patients with cholangiocarcinoma from other countries, accounting for up to 29% of *FGFR2* rearrangements^26^, ^28^, ^31^; this fusion partner seems to be less frequent in Chinese patients (6.7% in our study and none in another study^30^), but definite conclusions could not be reached because of the limited sample size. Nonetheless, existing evidence does not support correlations of *FGFR2* fusion partners with tumor response to pemigatinib. Clinicogenomics analysis of FIGHT‐202 data did not show a difference in ORR or PFS between *FGFR2*‐*B1CC* and other partners, although concurrent genetic alterations in tumor suppressor genes in general and in *CDKN2A/B* and *TP53* correlated with a shorter PFS.^32^ The clinical implications of different *FGFR2* rearrangements in terms of prognosis and response to treatment need to be elucidated in further studies.

Differences in patient characteristics, such as age, ethnicity, proportion of patients with a history of hepatitis, and prior treatments, preclude a direct comparison of results from our study and FIGHT‐202.^26^ Nonetheless, despite a shorter median duration of treatment (3.8 vs 7.2 months), ORR (50.0% vs 35.5%) and DCR (100% vs 82.2%) in our study were numerically higher than those in FIGHT‐202, while no patients in our study achieved CR as compared with three patients in FIGHT‐202. Median PFS was comparable between the two studies (6.3 months in our study vs 6.9 months in FIGHT‐202). The median OS was not reached in our study, versus 21.1 months in FIGHT‐202. Although caution should also be taken in comparing our study with those of other FGFR inhibitors, tumor response to pemigatinib in our study was numerically better than that with infigratinib (ORR, 18.8%; DCR, 83.3%),^33^ derazantinib (ORR, 20.7%; DCR, 82.8%),^34^ and TAS‐120 (ORR, 25%; DCR, 79%)^35^ for cholangiocarcinoma carrying *FGFR2* fusions or rearrangements in predominantly white populations. Whether Asian patients have a better response to this class of drugs remains to be determined. We will continue to evaluate the efficacy of pemigatinib based on mature OS data.

TEAEs were generally mild in Chinese patients with cholangiocarcinoma receiving pemigatinib. TEAEs of ≥grade 3, leading to dose reduction, dose interruption, and treatment discontinuation occurred in eight (25.8%), one (3.2%), four (12.9%), and zero patients, respectively, as compared with 93 (64%), 20 (14%), 62 (42%), and 13 (9%) in the other patient populations in FIGHT‐202.^26^ The commonly reported adverse events for *FGFR* inhibitors, including hyperphosphatemia, hypophosphatasemia, ocular disorders, and nail toxicities, were mostly <grade 3, with two patients having ≥grade 3 events. In particular, the management of hyperphosphatemia usually involves dose modification or interruption; however, this was not required given the mild severity of hyperphosphatemia events in our study. Neither was hypophosphatasemia a safety concern in our study, whereas 12% of patients had ≥grade 3 hypophosphatasemia in FIGHT‐202. The shorter duration of treatment and follow‐up and differences in patient characteristics may have resulted in better tolerability findings in our study versus FIGHT‐202.

NGS‐based assays provide comprehensive and precise profiling of genetic alterations, which is valuable for identifying predictive biomarkers and resistance mechanisms. Foundation One CDx has been approved by the US Food and Drug Administration as a companion diagnostic for pemigatinib.^36^ In China, an NGS‐based companion diagnostic for pemigatinib (Malignant Neoplasms Multi‐Gene Analysis Kit) is being developed in parallel, to be used for identifying patients with cholangiocarcinoma carrying *FGFR2* rearrangements who are eligible for pemigatinib treatment.

Because of the lack of an efficacious second‐line standard of care for cholangiocarcinoma, this phase II study did not include a comparator arm. Another limitation of this study was a sample size of 31 enrolled patients as compared with 107 patients with *FGFR2* rearrangements included in FIGHT‐202,^26^ because patient accrual in a single country usually takes a long time for rare cancers. Durations of treatment and follow‐up were relatively short as of the data cutoff, limiting time‐to‐event and safety assessments; the mature OS data will provide additional evidence for efficacy of pemigatinib in Chinese patients with cholangiocarcinoma and *FGFR2* rearrangements.

In conclusion, the encouraging efficacy and favorable safety profile of pemigatinib support its usage as a treatment for Chinese patients with unresectable locally advanced or metastatic cholangiocarcinoma and *FGFR2* fusions or rearrangements who had progressed on at least one previous treatment. Owing to the promising results seen in previously treated patients, a phase III trial (FIGHT‐302; NCT03656536) conducted in multiple countries including China is currently ongoing to evaluate the efficacy and safety of pemigatinib versus gemcitabine plus cisplatin as a first‐line treatment for unresectable or metastatic cholangiocarcinoma with *FGFR2* rearrangements.

## AUTHOR CONTRIBUTIONS

All authors approved the submission of this manuscript and had full access to all of the data in the study. Guo‐Ming Shi, Xiao‐Yong Huang, Tian‐Fu Wen, Tan‐Qiang Song, and Ming Kuang contributed equally to this study. Guo‐Ming Shi, Xiao‐Yong Huang, Tian‐Fu Wen, Tan‐Qiang Song, Ming Kuang, Yang Luo, Yan Wang, Jia Fan, and Jian Zhou contributed to concept and design. Guo‐Ming Shi, Xiao‐Yong Huang, Tian‐Fu Wen, Tan‐Qiang Song, Ming Kuang, Jia Fan, Jian Zhou, and Yang Luo carried out drafting of the manuscript. Ming‐Xia Chen carried out statistical analysis. All authors were involved in patient enrollment, data acquisition and interpretation, responsibility for data accuracy, administrative, technical support, and critical revision of the manuscript.

## FUNDING INFORMATION

This study was funded by Innovent Biologics, Inc., Suzhou, Jiangsu, China.

## CONFLICT OF INTEREST

The authors declare no potential conflicts of interest.

## Supporting information


Figure S1
Figure S2
Table S1
Table S2
Table S3

## Data Availability

The anonymized data that support the findings of this study are available from the corresponding author upon reasonable request.
