# Supplementary material for: Pemigatinib in previously treated Chinese patients with locally advanced or metastatic cholangiocarcinoma carrying FGFR2 fusions or rearrangements: A phase II study
Source: Cancer Med. 2022 Sep 20;12(4):4137–46. doi: 10.1002/cam4.5273 (PMC9972033; doi:10.1002/cam4.5273)

## Supplementary materials

## Supplementary Table S1. Frequency of *FGFR2* rearrangements in Chinese patients with intrahepatic cholangiocarcinoma by study site in the molecular epidemiology study.

| **Study site** | **Investigator** | **Samples** | **Positive (N)** | **Positive rate (%)** |
| --- | --- | --- | --- | --- |
| Zhongshan Hospital, Fudan University | Jian Zhou | 172 (6)^*^ | 8 | 4.82 |
| Xin Hua Hospital Affiliated to Shanghai Jiao Tong University School of Medicine | Wei Gong | 12 | 1 | 8.33 |
| Tongji Hospital, Tongji Medical College of HUST | Bi-Xiang Zhang | 41 | 3 | 7.32 |
| The First Affiliated Hospital of Guangxi Medical University | Tao Peng | 40 | 3 | 7.50 |
| The First Affiliated Hospital of University of Science and Technology of China | Lian-Xin Liu | 139 (1)^*^ | 6 | 4.35 |
| West China Hospital, Sichuan University | Tian-Fu Wen | 52 (1)^*^ | 5 | 9.80 |
| Tianjin Cancer Hospital | Ti Zhang | 61 | 4 | 6.56 |
| The first hospital of Kunming | Jiang-Hua Ran | 26 (1)^*^ | 3 | 12.00 |
| The First Affiliated Hospital of Sun Yat-sen University | Ming Kuang | 40 | 1 | 2.50 |
| The Second Affiliated Hospital of Zhejiang University School of Medicine | Wei-Lin Wang | 40 | 3 | 7.50 |
| Shulan (Hangzhou) Hospital | Hai-Bo Mou | 9 | 0 | 0 |
| Hubei Cancer Hospital | Le-Qun Bao | 54 | 2 | 3.70 |
| Cancer Hospital of Chinese Academy of Medical Science | Hong Zhao | 42 (2)^*^ | 5 | 12.50 |
| Total | | 728 (11)^*^ | 44 | 6.14 |

* with positive *FGFR2* status report.

## Supplementary Table S2. Identified *FGFR2* fusion partners.

| ***FGFR2* variation** |  | **Pemigatinib 13.5 mg (*N* = 30)**  ***n* (%)** |
| --- | --- | --- |
| Fusion | FGFR2-WAC | 3 (10.0) |
|  | FGFR2-AHCYL1 | 2 (6.7) |
|  | FGFR2-BICC1 | 2 (6.7) |
|  | FGFR2-KIAA1598 | 2 (6.7) |
|  | FGFR2-AFF4 | 1 (3.3) |
|  | FGFR2-CCDC6 | 1 (3.3) |
|  | FGFR2-CTNNA3 | 1 (3.3) |
|  | FGFR2-DAAM1 | 1 (3.3) |
|  | FGFR2-EIF4ENIF1 | 1 (3.3) |
|  | FGFR2-EVI5 | 1 (3.3) |
|  | FGFR2-KIAA1524 | 1 (3.3) |
|  | FGFR2-PAWR | 1 (3.3) |
|  | FGFR2-PPHLN1 | 1 (3.3) |
|  | FGFR2-PSPC1 | 1 (3.3) |
|  | FGFR2-SORBS1 | 1 (3.3) |
|  | FGFR2-STAU1 | 1 (3.3) |
|  | FGFR2-TACC1 | 1 (3.3) |
|  | FGFR2-TACC2 | 1 (3.3) |
|  | FGFR2-TFAP2B | 1 (3.3) |
|  | FGFR2-TK1 | 1 (3.3) |
|  | FGFR2-TRIM15 | 1 (3.3) |
|  | FGFR2-UBP1 | 1 (3.3) |
|  | FGFR2-USO1 | 1 (3.3) |
|  | FGFR2-UTRN | 1 (3.3) |
|  | FGFR2-XPNPEP1 | 1 (3.3) |
| Fusion/Rearrangement | FGFR2-ID3 (intergenic) | 1 (3.3) |

## Supplementary Table S3. Primary and secondary efficacy endpoints assessed by the investigators.

|  | **EEP/PPS**  **(*N* = 30)** |
| --- | --- |
| Objective response rate *n* (%), 95% CI | 12 (40.0), 22.7–59.4 |
| Complete response | 0 |
| Partial response | 12 (40.0) |
| Stable disease | 17 (56.7) |
| Progressive disease | 1 (3.3) |
| Disease control rate, *n* (%), 95% CI | 29 (96.7), 82.8–99.9 |
| Time to response, median (95% CI), months | 2.1 (1.2–2.8) |
| Duration of response |  |
| Median (95% CI), months | NR (2.1–NE) |
| At 3 months, % (95% CI) | 83.3 (27.3–97.5) |
| Progression-free survival |  |
| Median (95% CI), months | NR (4.9–NE) |
| At 3 months, % (95% CI) | 96.7 (78.6–99.5) |
| At 6 months, % (95% CI) | 65.7 (33.5–85.1) |

Tumor response was assessed by the investigators according to RECIST v1.1. EEP=efficacy-evaluable population; PPS=per-protocol set; NR=not reached; NE=not evaluable.

## Supplementary Figure S1. Kaplan–Meier estimates of overall survival.


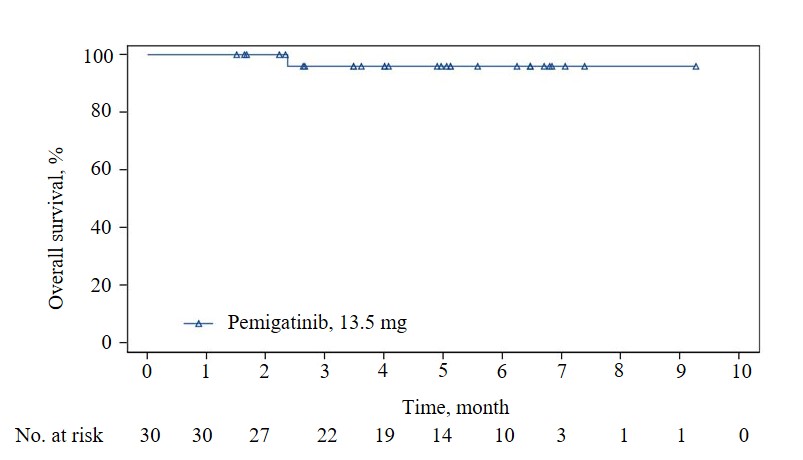


## Supplementary Figure S2. Change in mean serum concentrations of phosphate (A), calcium (B), 25-hydroxyvitamin D (C), and parathyroid hormone (D) over time.

A
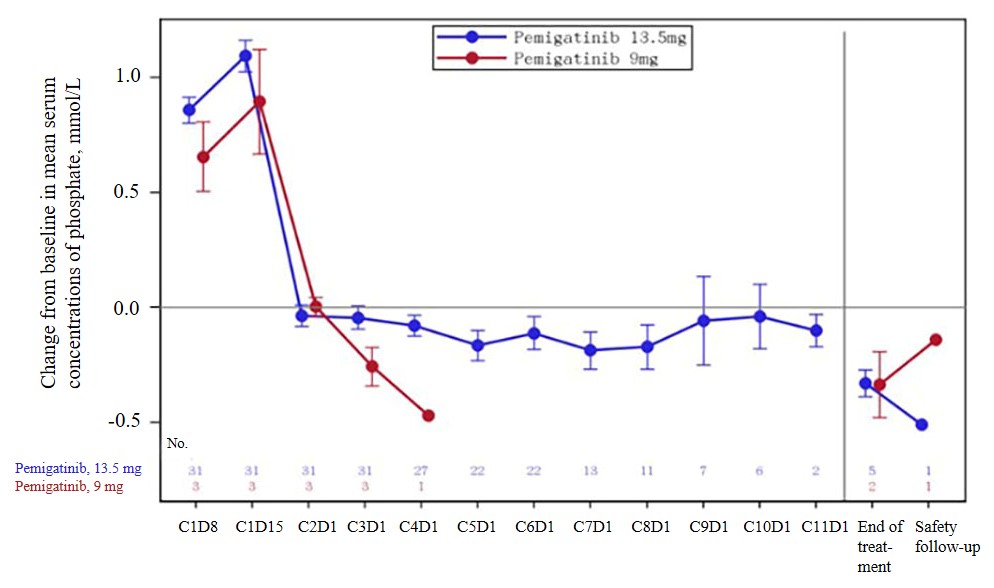


B
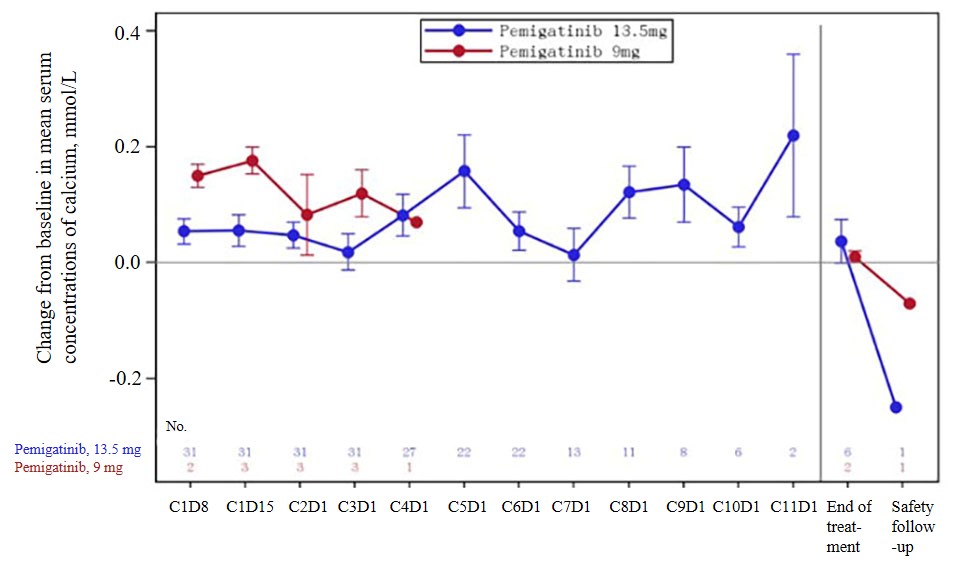


C
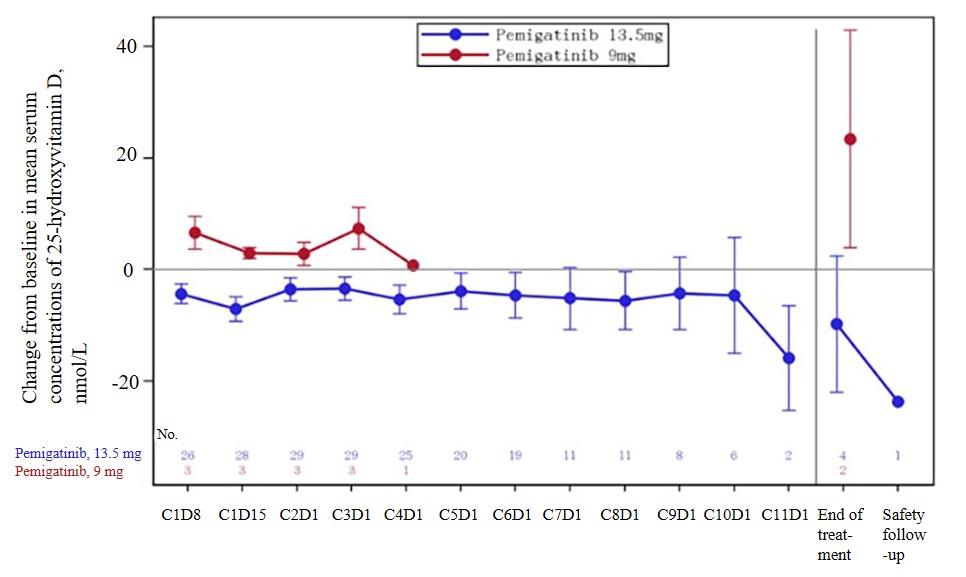


D
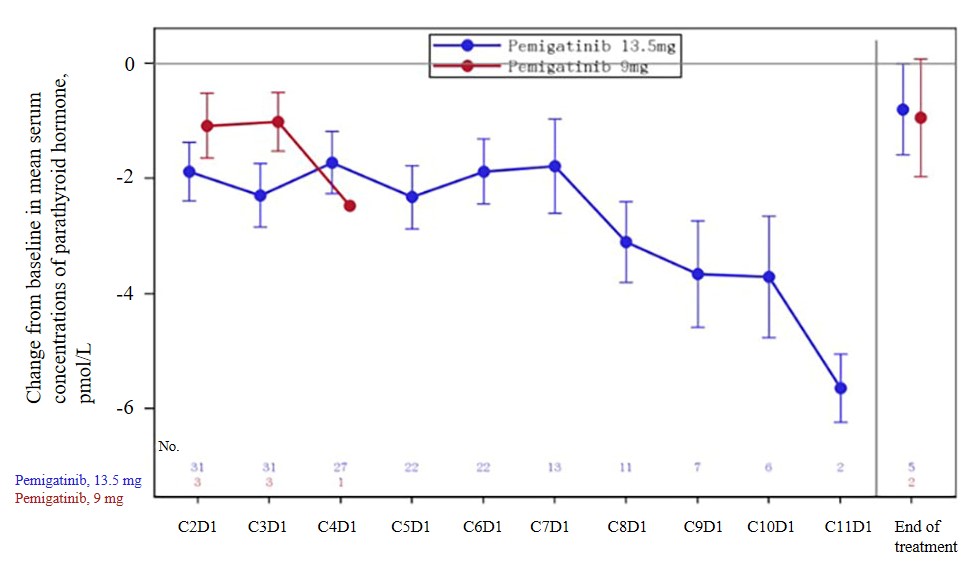

Supplement: Supplementary file 1 — Figure S1 Figure S2 Table S1 Table S2 Table S3 [file CAM4-12-4137-s001.docx]
